# Development of COPMAN-Air method for high-sensitivity detection of SARS-CoV-2 in air

**DOI:** 10.1038/s41598-025-99365-2

**Published:** 2025-04-24

**Authors:** Tomoyo Yoshinaga, Yoshinori Ando, Yumi Sato, Takeru Kishida, Masaaki Kitajima

**Affiliations:** 1 Shionogi & Co., Ltd., Osaka, Japan; 2 Kishida Clinic, Fukuoka, Japan; 3 Research Center for Water Environment Technology, School of Engineering, The University of Tokyo, Tokyo, Japan

**Keywords:** Air sampling, COPMAN, COPMAN-Air, Fever clinic, qPCR, SARS-CoV-2, Viral transmission, Public health, Air microbiology

## Abstract

Several studies have successfully detected SARS-CoV-2 in air samples. However, most of these studies focused on validating the air collection method, and there was no report on the development of a virus detection method. In this study, to detect viruses in air samples with greater sensitively than conventional detection methods, we utilized COPMAN, a highly sensitive virus detection method originally used for wastewater samples. We applied COPMAN to air samples, thereby developing COPMAN-Air. Briefly, this method efficiently detects the extremely low levels of viral RNA in air samples via three reaction steps: RT, preamplification, and qPCR, as it is performed with COPMAN. We evaluated COPMAN-Air using samples from a fever clinic for COVID-19 patients. COPMAN-Air demonstrated a higher detection rate of viral RNA compared with conventional methods, detecting the virus in 22 out of 23 samples (95.7%) vs. 14 out of 23 samples (60.9%). Additionally, a positive correlation (*r* = 0.70) was detected between the amount of viral RNA detected by COPMAN-Air and the number of confirmed COVID-19 cases, suggesting that COPMAN-Air could estimate the number of SARS-CoV-2-positive individuals in a given space based on the quantitative values of SARS-CoV-2 RNA in air samples. Surveillance systems for airborne pathogens using COPMAN-Air are expected to be valuable for estimating the number of infected individuals and for guiding the implementation of public health measures.

**Supplementary Information:**

The online version contains supplementary material available at 10.1038/s41598-025-99365-2.

## Introduction

SARS-CoV-2 is a virus that infects the human respiratory tract. It is widely accepted that this virus spreads through airborne transmission; therefore, one way to prevent its spread is to help people determine whether the air around them contains the virus^1–6^. Air sampling to detect the virus has been vigorously considered. Many of these studies have been conducted in health care settings, such as hospital isolation rooms for COVID-19 patients and quarantine hotels, where high concentrations of SARS-CoV-2-containing aerosols are expected^6–9^. However, considering the social implications of the virus, it is necessary to conduct a feasibility study in a more practical community setting. Some reports have successfully detected SARS-CoV-2 in public places such as student dormitories, schools, cafeterias, offices, shopping centres, airports, and public transport^9–12^. Nevertheless, because it is difficult to determine the number of SARS-CoV-2-infected individuals who have been or are currently present in such spaces during air sampling, it is not possible to accurately confirm the validity of the virus detection method. Furthermore, it cannot be determined whether the method is suitable for the early detection of infected individuals.

The primary method for detecting airborne SARS-CoV-2 is the quantitative measurement of viral RNA from an air sample via reverse transcription‒quantitative polymerase chain reaction (RT-qPCR)^8,9,11^. There have been many reports on air sampling methods^9,13^. However, since most of these reports have been unable to measure airborne SARS-CoV-2 quantitatively, there is a need to optimize the conventional protocol used after air sampling to improve the sensitivity of virus detection using RT-qPCR.

Recently, our group developed the COPMAN (COagulation and Proteolysis method using MAgnetic beads for nucleic acids in wastewater) method, which can detect SARS-CoV-2 RNA from wastewater samples with both high sensitivity and high throughput^14,15^. The amount of SARS-CoV-2 in the air, as well as that in wastewater, is likely significantly lower than that in clinical samples. Additionally, as we observed during the COVID-19 pandemic, once a pandemic occurs, the demand for air sample tests will increase markedly because room-by-room pathogen monitoring is needed in many facilities, such as hospitals, nursing homes, schools, hotels, and restaurants. There will be concerns about shortages of the resources necessary for virus testing, similar to the concerns that arose regarding clinical sample tests. The COPMAN method for wastewater testing is an inspection approach that allows near-full automation via LabDroid^15^. To achieve worthwhile social implementation, the protocol must be capable of handling a large number of air samples for virus monitoring in various spaces. To our knowledge, such efforts have not yet been sufficiently verified.

In this study, we developed COPMAN-Air, a new method that applies near-full-automation-available COPMAN technology, and investigated the detection sensitivity of SARS-CoV-2 RNA from air samples obtained from a Thermo Fisher Scientific AerosolSense Sampler. Some reports have shown that this active air sampler is useful in collecting SARS-CoV-2-containing aerosols. However, considering the reported sensitivity and the throughput estimated from the method used, there is still room for improvement in the detection method for social implementation. Furthermore, using our method, we measured the amount of SARS-CoV-2 RNA in air samples collected at a so-called “fever clinic,” where outpatients with cold symptoms were examined.

## Results

### Development of the COPMAN-Air method

**Fig. 1:**
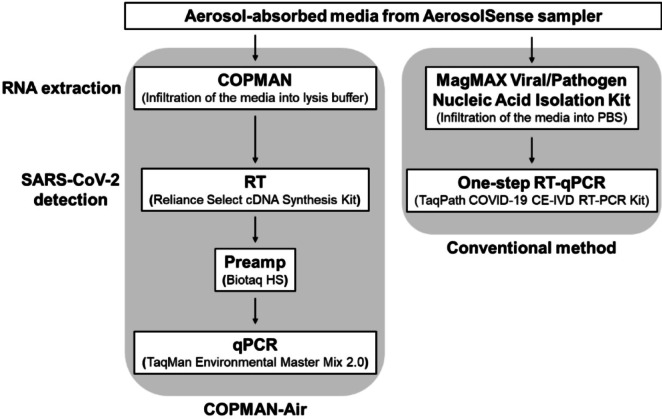
Diagram of the hierarchical experimental design. COPMAN-Air and conventional methods are shown in the left and right panels, respectively.

We developed a novel approach (COPMAN-Air) that integrates RNA extraction using the COPMAN method from aerosol-absorbing media collected by the AerosolSense sampler with virus detection via the RT-Preamplification (Preamp)-qPCR method. This approach allows for the quantitative measurement of SARS-CoV-2 RNA in air samples (Fig. 1). Additionally, we confirmed its ability to detect SARS-CoV-2 RNA from a sampler spiked with inactivated SARS-CoV-2 using the COPMAN-Air method.

**Fig. 2:**
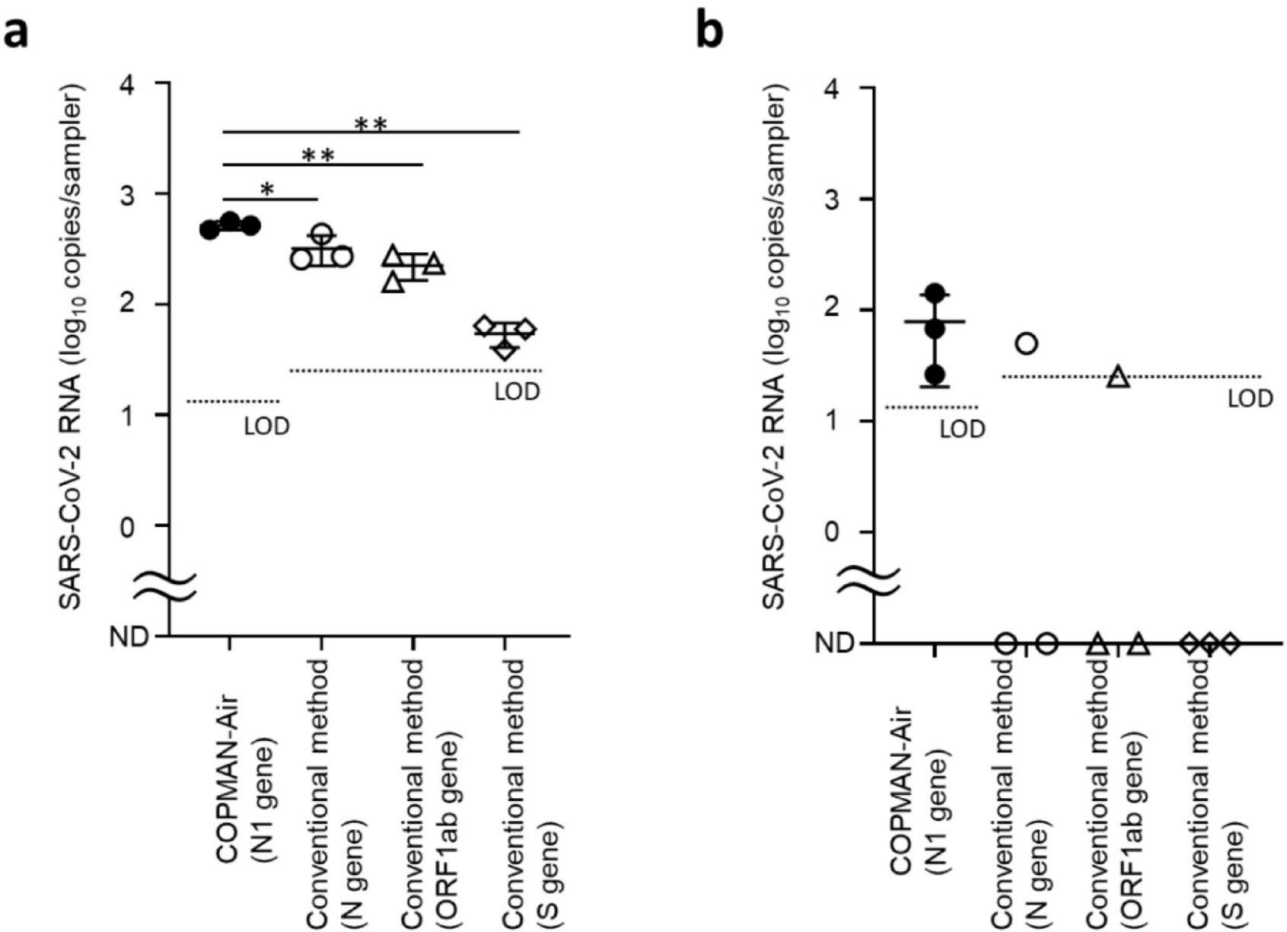
Comparative verification of COPMAN-Air and the conventional method using viral-spiked samples. SARS-CoV-2 RNA is shown when (**a**) 50 and (**b**) 1000 copies of heat-inactivated SARS-CoV-2 viruses were spiked and recovered from the sampler via the COPMAN-Air or conventional method. Each bar represents the mean and standard deviation when viral RNA was detected in 3/3 of the samples. ND is indicated when no signal was detected or when the signal was detected below the LOD. If viral RNA was detected in only one or two out of three samples, only the respective values are displayed. *P* values were calculated using an unpaired t test for the COPMAN-Air method vs. the conventional method per spiked copy of heat-inactivated SARS-CoV-2 (*p < 0.05, **p < 0.01).

To evaluate the sensitivity of COPMAN-Air, we adopted a previously reported method as the conventional method and used it as a reference for comparison with COPMAN-Air^11^. We assessed its ability to detect and quantify SARS-CoV-2 RNA from media spiked with 50, 100, and 1000 copies of inactivated SARS-CoV-2. The theoretical limit of detection (LOD) of the COPMAN-Air test was lower than that of conventional methods, which use 5 µL of RNA, as 14 µL of RNA is subjected to qPCR detection with the COPMAN-Air method. We compared the observed concentrations between the two methods since both methods were able to detect quantifiable amounts of SARS-CoV-2 RNA from media spiked with 1,000 copies of the virus. COPMAN-Air resulted in a higher observed concentration (516.2 copies/sampler) than the conventional method did (319.5 copies/sampler (N gene), 224.1 copies/sampler (ORF1ab gene), 54.1 copies/sampler (S gene)). A t test was conducted to compare COPMAN-Air with the conventional method (N gene) and revealed a statistically significant difference (*p* < 0.05). (Fig. 2a).

Compared with the conventional method, the COPMAN-Air method exhibited greater accuracy, with a coefficient of variation of 7.2%, whereas those of the conventional methods were 24.9% (N gene), 21.7% (ORF1ab gene), and 20.3% (S gene).

When spiked with 50 copies of the virus, which is close to the LOD level, COPMAN-Air exhibited a greater detection rate than the conventional method did (Fig. 2b). COPMAN-Air can be considered a more sensitive method than the conventional method due to its lower theoretical LOD and greater observed concentrations and detection rates.

### Validation of COPMAN-Air and its comparison with the conventional method at a fever clinic

**Fig. 3:**
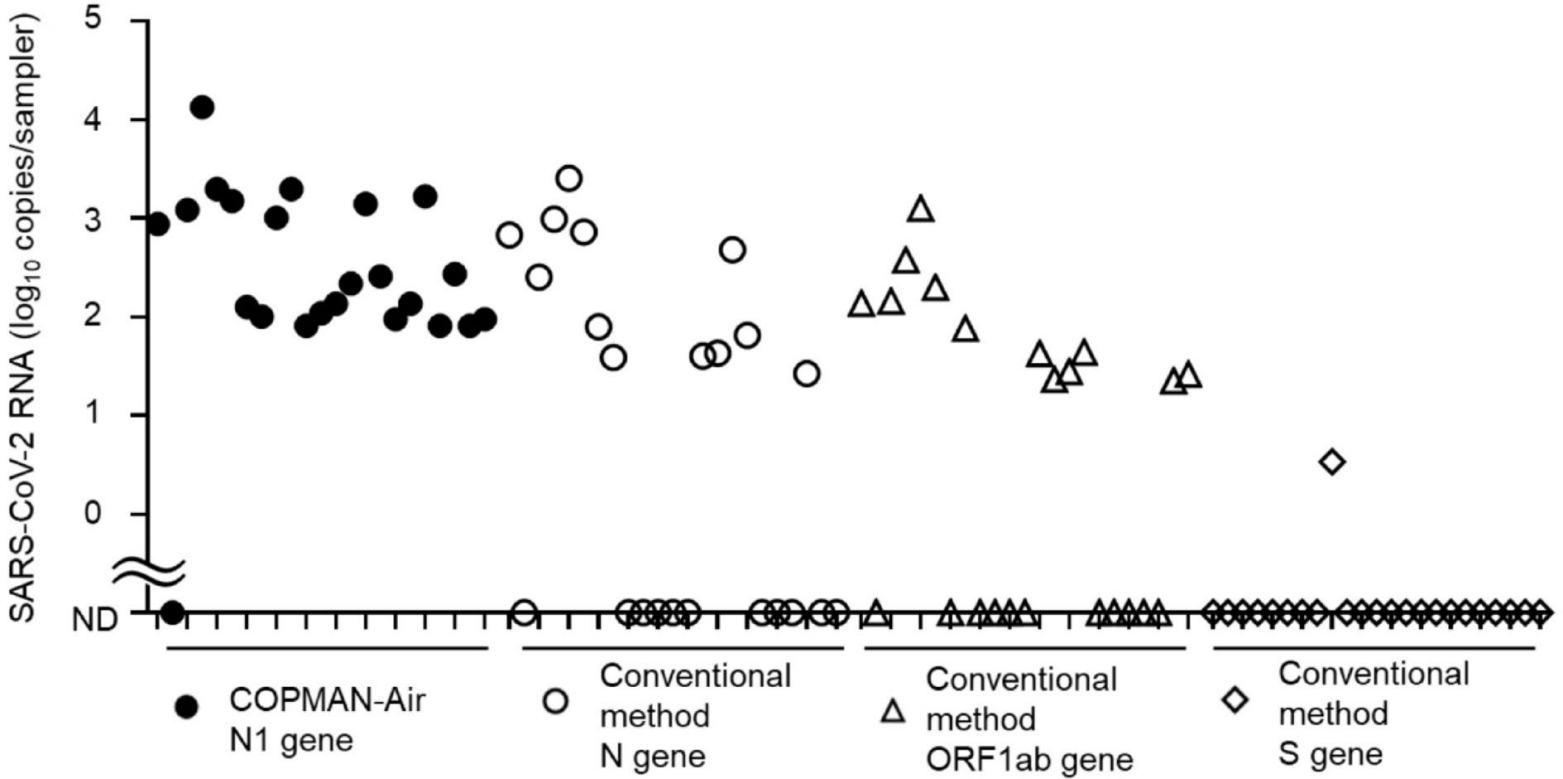
Comparative verification of the COPMAN-Air and conventional methods using samples from the fever clinic. SARS-CoV-2 RNA was measured via the COPMAN method (N1 gene, filled circle) or conventional methods (N gene, open circle; ORF1ab gene, open triangle; S gene, open diamond) using 23 samples obtained in duplicate from the fever clinic.

To compare COPMAN-Air with the conventional method using field aerosol samplers, we conducted air sampling at a fever clinic during the 5th wave of COVID-19 infection in Japan and evaluated these samples. Based on the measured results, COPMAN-Air was able to detect SARS-CoV-2 more accurately in 22 (95.7%) out of 23 samples, with a mean concentration of 1217 copies/sampler, whereas the conventional method detected the virus in only 14 (60.9%) out of 23 samples (Fig. 3). These findings from the clinical experiments led us to conclude that COPMAN-Air demonstrated superior detection sensitivity than the conventional method did. As a result, we decided to use COPMAN in our future clinical experiments.

### Correlation analysis of SARS-CoV-2 in air samples and the number of COVID-19 patients

**Fig. 4:**
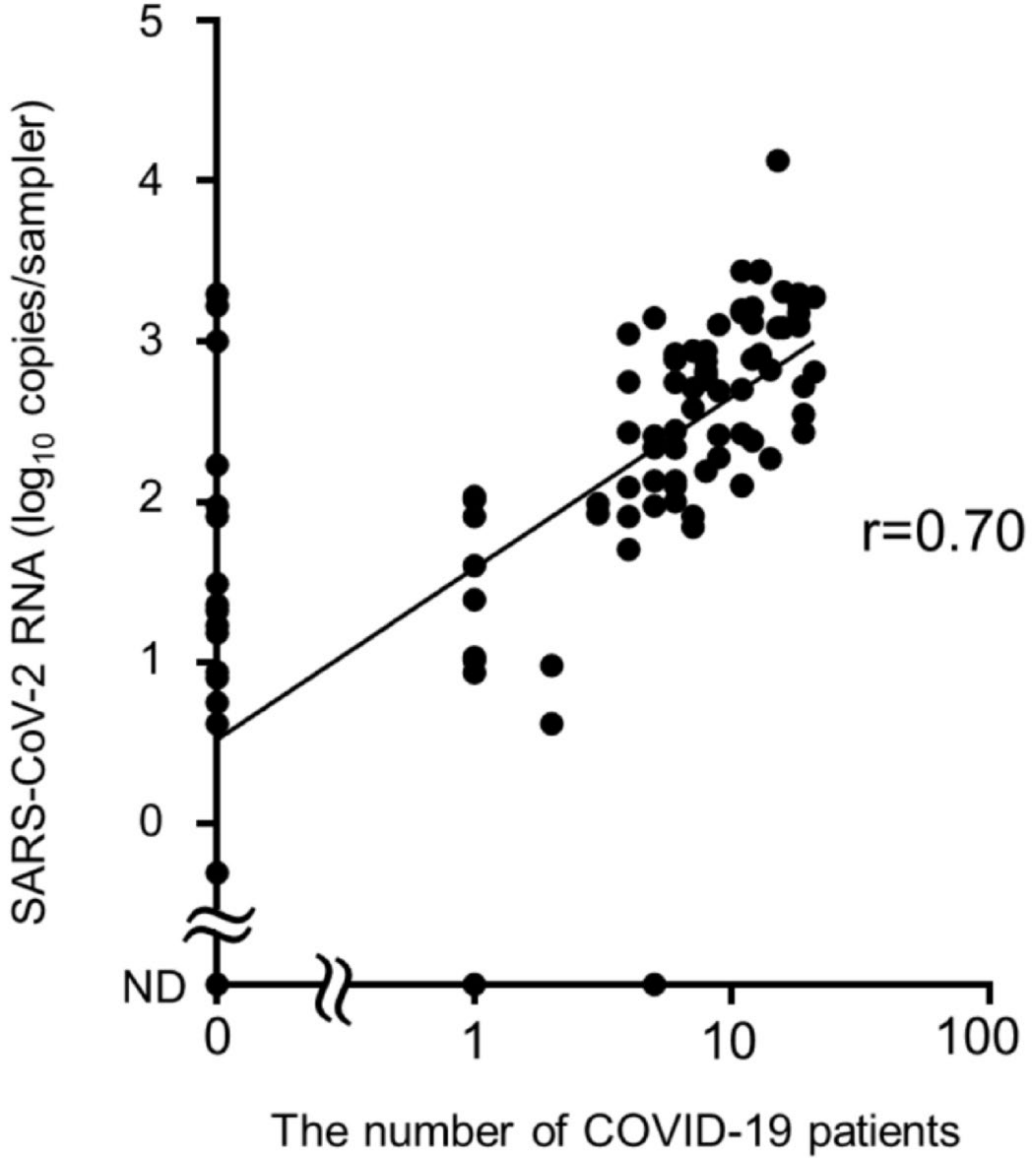
Correlation between the number of COVID-19 patients and the amount of SARS-CoV-2 RNA in air samples. A scatter plot shows the amount of SARS-CoV-2 RNA in air samples collected via the COPMAN-Air method and the number of COVID-19 patients. A correlation analysis was performed using Pearson’s correlation test.

COPMAN-Air could detect SARS-CoV-2 even when only a few COVID-19 patients were present (Fig. 3 and Supplementary Table S1). To compare this with the total number of patients, we conducted extensive additional air sampling at the fever clinic during the 6th and 7th waves of COVID-19 infection in Japan (Supplementary Table S1). We evaluated the correlation between the number of COVID-19 patients and the amount of SARS-CoV-2 RNA detected in all air samples via COPMAN-Air (Fig. 4). The data points plotted on the scatter plot closely aligned along an approximately straight line (y = 1.066x + 1.590), suggesting a linear relationship between the number of COVID-19 patients and the amount of viral RNA detected in the air samples. The results of the Pearson correlation test revealed a positive correlation between the number of COVID-19 patients and the number of SARS-CoV-2 RNA copies in the air samples measured via COPMAN-Air (*r* = 0.70).

## Discussion

For most of the air samples available in this study, we were able to obtain quantitative data for SARS-CoV-2 RNA via COPMAN-Air. There are two possible reasons why COPMAN-Air showed a higher observed concentration and a lower theoretical LOD than the conventional method did. Although the protocols differ significantly, the most influential factor contributing to these differences is the nucleic acid extraction step from the aerosol-absorbed media. In the conventional method, the media is infiltrated into PBS; however, virus particles suspended in PBS may be reabsorbed onto the media. In contrast, in the COPMAN-Air approach, the media is infiltrated into lysis buffer; therefore, the virus particles are completely destroyed and cannot adsorb onto the media again. Consequently, it is assumed that viral RNA can be extracted from media more efficiently. In addition, it is not difficult to imagine that the amount of SARS-CoV-2 in air samples is extremely low compared with that in nasal swabs and saliva samples from COVID-19 patients. With this in mind, we incorporated a preamp step into both the COPMAN-Air and COPMAN methods for wastewater samples to increase the equivalent RNA volume introduced during the qPCR detection step, thereby improving the detection sensitivity of viral RNA^14–16^.

In this study, we conducted air sampling in a fever clinic, a setting that can be described as an intermediate level between a hospital setting for COVID-19 inpatients and a community setting where an indefinite number of infected individuals visit temporarily. Furthermore, the setting involves a mix of COVID-19-confirmed individuals and SARS-CoV-2-uninfected individuals. As shown in Fig. 4, we detected a positive correlation between the amount of SARS-CoV-2 RNA in air samples and the number of COVID-19 patients in this setting. These data suggest that as the number of individuals excreting SARS-CoV-2-containing aerosols increases, the amount of viral RNA in the air sample also increases; this allowed us to verify the correlation between the number of COVID-19 patients and the amount of SARS-CoV-2 RNA in air samples. In our data obtained from the fever clinic, while viral RNA was detected in air samples from one COVID-19 patient, it was also detected during periods when no COVID-19 patients were present. Two possible explanations for this can be considered. First, because this fever clinic does not perform a complete cleanup every day, it is possible that virus particles from the previous day may have adhered to the floor or equipment as droplets and were then reaerosolized and detected once they were resuspended in the air. Second, in addition to patients with cold symptoms, doctors and nurses are also present in the fever clinic, and some of them might have been asymptomatically infected since they were not tested daily for SARS-CoV-2. In other words, it is possible that not all the viral RNA detected in this setting was from COVID-19 patients who were present during air sampling.

This study has several limitations that warrant consideration. First, the combination of COPMAN-Air with other air sampling methods is not yet available. In many previous reports, air collection methods, such as cyclones, filters, impactors, impingers, and water-based condensations have been adopted, and the sampling volume, flow rate, sampling time, and collection medium of each method have been studied in detail^9,13^. The AerosolSense sampler was determined suitable for use in this study because previous works have reported its ability to detect SARS-CoV-2 RNA, and it was assumed that it would be feasible to combine it with the COPMAN approach^11,17,18^. Second, asymptomatically infected individuals, who play an important role in viral transmission in community settings^19–22^, are not completely included, so the setting of this study does not fully mimic the community setting. Third, other environmental factors, especially the variation in temperature and humidity levels of each public space and the influence of air flow caused by air conditioning and ventilation systems, could not be considered in this study. Fourth, although we clearly confirmed a positive correlation between airborne SARS-CoV-2 RNA and the number of SARS-CoV-2-positive individuals, the amount of SARS-CoV-2 excreted by individuals was not considered in this analysis. By including this parameter in the correlation analysis, we anticipate an increase in the precision of estimating the number of virus-positive individuals based on airborne virus concentration as an indicator. These are factors to be addressed in the future.

Taken together, the data reported here strongly suggest that highly sensitive detection methods targeting airborne viruses could be advantageous for monitoring air conditions to prevent aerosol transmission. The quantitative values of viral RNA in air samples could be used to estimate the number of infected individuals who have been present in each space, and air sample tests have the potential to complement standard clinical tests. This approach may also be applicable to other viruses, such as influenza and respiratory syncytial viruses, that are transmitted between humans via aerosols and/or air. These viruses spread seasonally; however, if a new strain emerges with a mutation that increases its virulence or reduces the effectiveness of available vaccines and drugs, it is not difficult to imagine that such viruses, such as SARS-CoV-2, could spread quickly around the world. Finally, compared with clinical testing intended for individuals, this air sample test can represent multiple people in one sample, rendering it cost-effective and similar to other environmental tests. Based on these findings, surveillance systems for airborne pathogens are expected to function as key public health measures that help establish a society that is resilient against the next pandemic.

## Materials and methods

### Preparation of inactivated SARS-CoV-2

An isolated SARS-CoV-2 strain (hCoV-19/Japan/TY-WK-521/2020, GISAID Accession ID: EPI_ISL_408667) was provided by the National Institute of Infectious Diseases, Japan. SARS-CoV-2 was propagated in VeroE6-TMPRSS2 cells (JCRB1819)^23^, and the virus was inactivated by heating at 65 °C for 30 min^24^. After inactivation, the viral mixture was aliquoted and stored at -80 °C. RNA was extracted from the inactivated virus via a QIAamp Viral RNA Mini Kit (QIAGEN, Hilden, Germany). The viral RNA was quantified via the same method as described for COPMAN-Air. The copy number of the stock solution was 1.58 × 10^5^ copies/µL.

### Viral quantification from air samples via COPMAN-Air

The newly developed COPMAN-Air method consists of sample collection via an AerosolSense sampler (Thermo Fisher Scientific, Waltham, MA, USA), followed by RNA extraction, RT, preamp, and qPCR via the COPMAN method^14,15^.

The AerosolSense sampler, capable of sustained sampling over a long period of time, was used for air sampling. The RNA was then purified via the COPMAN method with AerosolSense cartridges (Thermo Fisher Scientific). Briefly, after aerosol-absorbed media were infiltrated with lysis buffer, including DTT and proteinase, the mixture was removed and heated at 56 °C for 10 min. Crude RNA was extracted with phenol/chloroform/isoamyl alcohol (25:24:1) and then purified with magnetic beads to obtain an RNA extract. Viral RNA was quantified via the COPMAN method. An aliquot of 14 µL of total RNA was subjected to cDNA synthesis via the Reliance Select cDNA synthesis kit (Bio-Rad Laboratories, Hercules, CA, USA) under the following conditions: 50 °C for 60 min and then 95 °C for 1 min in a 20-µL reaction mixture with 2 pmol of reverse primer of SARS-CoV-2 (N1 gene). The resulting SARS-CoV-2 cDNAs were preamplified for 10 cycles via Biotaq HS (Bioline Reagents Ltd., London, UK) under the following conditions: 95 °C for 10 min; 10 cycles at 95 °C for 15 s; 55 °C for 15 s; and 72 °C for 30 s, in a 30-µL volume of reaction mixture containing 9 pmol of each forward and reverse primers for SARS-CoV-2 (N1 gene). Finally, 2.5 µL of the preamplified product of SARS-CoV-2 was quantified via qPCR using the TaqMan Environmental Master Mix 2.0 (Thermo Fisher Scientific) under the following conditions: 95 °C for 10 min; 45 cycles of 95 °C for 15 s; and 60 °C for 30 s in a 20-µL singleplex reaction mixture containing 10 pmol each of the reverse and forward primers and 7.5 pmol of the TaqMan probe.

### Viral quantification from air samples according to the conventional method

The conventional method consists of sample collection using the AerosolSense sampler, followed by RNA extraction using the MagMAX Viral/Pathogen Nucleic Acid Isolation Kit (Thermo Fisher Scientific) and subsequent quantification via RT‒qPCR. RNA purification from the AerosolSense cartridges was carried out using the MagMAX Viral/Pathogen Nucleic Acid Isolation Kit. Briefly, after the aerosol-absorbed media were infiltrated with PBS, extracted, and mixed with proteinase K (ProK) and total nucleic acid magnetic beads, the mixture was heated at 65 °C for 5 min. A final RNA sample was eluted from the beads after washing. The RNA was quantified by RT-PCR using a TaqPath COVID-19 CE-IVD RT-PCR Kit (Thermo Fisher Scientific) according to the manufacturer’s instructions under the following conditions: 25 °C for 2 min, 53 °C for 10 min, 95 °C for 2 min, and 40 cycles of 95 °C for 3 s and 60 °C for 30 s in a 25-µL reaction mixture containing three primer/probe sets specific to different SARS-CoV-2 genomic regions (open reading frame 1ab (ORF1ab), spike (S) protein and nucleocapsid (N) protein-encoding genes).

### Comparison of the COPMAN-Air and conventional methods

The aerosol-absorbing media of the AerosolSense cartridges were spiked with 50, 100, or 1000 copies of inactivated SARS-CoV-2 in triplicate experiments. Nucleic acids from the cartridges were extracted via the COPMAN-Air or conventional methods. The target viral genes were subsequently quantified via each method. The LOD was calculated by assuming that each method could detect as few as one copy of target cDNA in a qPCR. The mean and standard deviation were calculated when viral RNA was detected in all 3 samples. The P values were calculated via an unpaired t test comparing the COPMAN-Air and conventional methods for each spiked copy of heat-inactivated SARS-CoV-2.

### Air sampling

The airborne aerosols were sampled at a fever clinic in Fukuoka City, Japan. This fever clinic is intended for use by all outpatients with cold symptoms, including those of COVID-19. COVID-19 diagnosis is determined by a doctor’s examination and/or a clinical test. The sampling was carried out by placing AerosolSense samplers with AerosolSense cartridges at two locations in the fever clinic. One sampler was positioned on the floor (Point A), while the other was placed on a low table, approximately 30 centimetres above the floor (Point B). Sampling was performed in July and September 2022 (during the 5th wave of COVID-19 infection in Japan), in March (during the 6th wave), and in July and August 2023 (during the 7th wave). The sampling durations ranged from approximately 3 to 10 h. Depending on the time, during or after the peak period of COVID-19, the number of positive cases per day varied from zero to several dozen. In addition, outpatients, unlike inpatients, who had visited the clinic for less than one hour, included SARS-CoV-2-infected individuals (Supplementary Table S1). The amount of SARS-CoV-2 RNA measured by the COPMAN-Air in each air sample, relative to the corresponding number of COVID-19 patients during each sampling period, was plotted (Fig. 4), and a correlation analysis was performed using Pearson’s correlation test.

### Statistical analysis

All the statistical analyses were performed via GraphPad Prism 8.4.3.

## Electronic supplementary material

Below is the link to the electronic supplementary material.

Supplementary Material 1

## Data Availability

The authors confirm that the data supporting the findings of this study are available within this article and its Supplementary Information files.
