# Supplementary material for: Development of COPMAN-Air method for high-sensitivity detection of SARS-CoV-2 in air
Source: Sci Rep. 2025 Apr 24;15:14340. doi: 10.1038/s41598-025-99365-2 (PMC12022069; doi:10.1038/s41598-025-99365-2)
Supplement: Supplementary file 1 — Supplementary Material 1 [file 41598_2025_99365_MOESM1_ESM.docx]

# Supplementary Information

## Title

Development of COPMAN-Air method for high-sensitivity detection of SARS-CoV-2 in air

## Author information

Tomoyo Yoshinaga^1^, Yoshinori Ando^1^*, Yumi Sato^1^, Takeru Kishida^2^, and Masaaki Kitajima^3^

1 Shionogi & Co., Ltd., Osaka, Japan

2 Kishida Clinic, Fukuoka, Japan

3 Research Center for Water Environment Technology, School of Engineering, The University of Tokyo, Tokyo, Japan

*Corresponding author

Yoshinori Ando

E-mail: [yoshinori.ando@shionogi.co.jp](mailto:yoshinori.ando@shionogi.co.jp)

## **Supplementary Table S1. Detail information of air sampling and outpatients at the fever clinic**

| Sampling  date | Sampling  time  (hours) | Sampling  point | COVID-19  wave in Japan | Number of  diagnostic  tests | Number of  COVID-19  patients |
| --- | --- | --- | --- | --- | --- |
| 23/Jul/2022 | 4 | B | 5th wave | 10 | 7 |
| 23/Jul/2022 | 3 | B | 5th wave | 3 | 1 |
| 25/Jul/2022 | 10 | A | 5th wave | 24 | 15 |
| 25/Jul/2022 | 10 | B | 5th wave | 24 | 15 |
| 26/Jul/2022 | 10 | A | 5th wave | 23 | 18 |
| 26/Jul/2022 | 10 | B | 5th wave | 23 | 18 |
| 27/Jul/2022 | 10 | A | 5th wave | 16 | 6 |
| 27/Jul/2022 | 10 | B | 5th wave | 16 | 6 |
| 3/Sep/2022 | 3 | A | 5th wave | 3 | 0 |
| 3/Sep/2022 | 3 | B | 5th wave | 3 | 0 |
| 3/Sep/2022 | 3 | A | 5th wave | 4 | 1 |
| 3/Sep/2022 | 3 | B | 5th wave | 4 | 1 |
| 5/Sep/2022 | 10 | A | 5th wave | 10 | 6 |
| 5/Sep/2022 | 10 | B | 5th wave | 10 | 6 |
| 7/Sep/2022 | 10 | A | 5th wave | 15 | 5 |
| 7/Sep/2022 | 10 | B | 5th wave | 15 | 5 |
| 8/Sep/2022 | 10 | A | 5th wave | 10 | 5 |
| 8/Sep/2022 | 10 | B | 5th wave | 10 | 5 |
| 9/Sep/2022 | 10 | A | 5th wave | 3 | 0 |
| 9/Sep/2022 | 10 | B | 5th wave | 3 | 0 |
| 10/Sep/2022 | 7.5 | A | 5th wave | 11 | 4 |
| 10/Sep/2022 | 3 | B | 5th wave | 10 | 4 |
| 10/Sep/2022 | 3 | B | 5th wave | 1 | 0 |
| 11/Mar/2023 | 2 | B | 6th wave | 0 | 0 |
| 11/Mar/2023 | 2 | A | 6th wave | 0 | 0 |
| 11/Mar/2023 | 2 | B | 6th wave | 0 | 0 |
| 11/Mar/2023 | 2 | A | 6th wave | 0 | 0 |
| 11/Mar/2023 | 2 | B | 6th wave | 0 | 0 |
| 11/Mar/2023 | 2 | A | 6th wave | 0 | 0 |
| 13/Mar/2023 | 10 | B | 6th wave | 11 | 0 |
| 13/Mar/2023 | 10 | A | 6th wave | 11 | 0 |
| 14/Mar/2023 | 10 | B | 6th wave | 5 | 0 |
| 14/Mar/2023 | 10 | A | 6th wave | 5 | 0 |
| 15/Mar/2023 | 10 | B | 6th wave | 4 | 1 |
| 15/Mar/2023 | 10 | A | 6th wave | 4 | 1 |
| 16/Mar/2023 | 10 | B | 6th wave | 7 | 1 |
| 16/Mar/2023 | 10 | A | 6th wave | 7 | 1 |
| 17/Mar/2023 | 10 | B | 6th wave | 2 | 0 |
| 17/Mar/2023 | 10 | A | 6th wave | 2 | 0 |
| 18/Mar/2023 | 10 | B | 6th wave | 3 | 0 |
| 18/Mar/2023 | 10 | A | 6th wave | 3 | 0 |
| 20/Mar/2023 | 10 | B | 6th wave | 9 | 3 |
| 20/Mar/2023 | 10 | A | 6th wave | 9 | 3 |
| 22/Mar/2023 | 10 | B | 6th wave | 10 | 0 |
| 22/Mar/2023 | 10 | A | 6th wave | 10 | 0 |
| 23/Mar/2023 | 10 | B | 6th wave | 7 | 2 |
| 23/Mar/2023 | 10 | A | 6th wave | 7 | 2 |
| 24/Mar/2023 | 10 | B | 6th wave | 4 | 0 |
| 24/Mar/2023 | 10 | A | 6th wave | 4 | 0 |
| 25/Mar/2023 | 5 | B | 6th wave | 0 | 0 |
| 25/Mar/2023 | 5 | A | 6th wave | 0 | 0 |
| 25/Mar/2023 | 4 | B | 6th wave | 4 | 1 |
| 25/Mar/2023 | 4 | A | 6th wave | 4 | 1 |
| 24/Jul/2023 | 4 | B | 7th wave | 25 | 19 |
| 24/Jul/2023 | 4 | A | 7th wave | 25 | 19 |
| 24/Jul/2023 | 4 | B | 7th wave | 9 | 4 |
| 24/Jul/2023 | 4 | A | 7th wave | 9 | 4 |
| 25/Jul/2023 | 10 | B | 7th wave | 19 | 13 |
| 25/Jul/2023 | 10 | A | 7th wave | 19 | 13 |
| 26/Jul/2023 | 10 | B | 7th wave | 30 | 19 |
| 26/Jul/2023 | 10 | A | 7th wave | 30 | 19 |
| 27/Jul/2023 | 10 | B | 7th wave | 21 | 12 |
| 27/Jul/2023 | 10 | A | 7th wave | 21 | 12 |
| 28/Jul/2023 | 10 | B | 7th wave | 20 | 11 |
| 28/Jul/2023 | 10 | A | 7th wave | 20 | 11 |
| 29/Jul/2023 | 10 | B | 7th wave | 25 | 14 |
| 29/Jul/2023 | 10 | A | 7th wave | 25 | 14 |
| 31/Jul/2023 | 10 | B | 7th wave | 34 | 21 |
| 31/Jul/2023 | 10 | B | 7th wave | 34 | 21 |
| 1/Aug/2023 | 10 | B | 7th wave | 17 | 7 |
| 1/Aug/2023 | 10 | A | 7th wave | 17 | 7 |
| 2/Aug/2023 | 10 | B | 7th wave | 22 | 13 |
| 2/Aug/2023 | 10 | A | 7th wave | 22 | 13 |
| 3/Aug/2023 | 10 | B | 7th wave | 29 | 9 |
| 3/Aug/2023 | 10 | A | 7th wave | 29 | 9 |
| 4/Aug/2023 | 10 | B | 7th wave | 20 | 11 |
| 4/Aug/2023 | 10 | A | 7th wave | 20 | 11 |
| 5/Aug/2023 | 10 | B | 7th wave | 23 | 4 |
| 5/Aug/2023 | 10 | A | 7th wave | 23 | 4 |
| 7/Aug/2023 | 10 | B | 7th wave | 21 | 16 |
| 7/Aug/2023 | 10 | A | 7th wave | 21 | 16 |
| 8/Aug/2023 | 10 | B | 7th wave | 20 | 6 |
| 8/Aug/2023 | 10 | A | 7th wave | 20 | 6 |
| 9/Aug/2023 | 6.5 | B | 7th wave | 13 | 5 |
| 9/Aug/2023 | 6.5 | A | 7th wave | 13 | 5 |
| 10/Aug/2023 | 8 | B | 7th wave | 15 | 8 |
| 10/Aug/2023 | 8 | A | 7th wave | 15 | 8 |
| 16/Aug/2023 | 6.5 | B | 7th wave | 20 | 11 |
| 16/Aug/2023 | 6.5 | A | 7th wave | 20 | 11 |
| 17/Aug/2023 | 10 | B | 7th wave | 25 | 9 |
| 17/Aug/2023 | 10 | A | 7th wave | 25 | 9 |
| 18/Aug/2023 | 10 | B | 7th wave | 19 | 12 |
| 18/Aug/2023 | 10 | A | 7th wave | 19 | 12 |
| 19/Aug/2023 | 10 | B | 7th wave | 22 | 7 |
| 19/Aug/2023 | 10 | A | 7th wave | 22 | 7 |
| 21/Aug/2023 | 10 | B | 7th wave | 34 | 18 |
| 21/Aug/2023 | 10 | A | 7th wave | 34 | 18 |
| 22/Aug/2023 | 10 | B | 7th wave | 20 | 8 |
| 22/Aug/2023 | 10 | A | 7th wave | 20 | 8 |
| 23/Aug/2023 | 10 | B | 7th wave | 32 | 11 |
| 23/Aug/2023 | 10 | A | 7th wave | 32 | 11 |
| 24/Aug/2023 | 10 | B | 7th wave | 23 | 8 |
| 24/Aug/2023 | 10 | A | 7th wave | 23 | 8 |
| 25/Aug/2023 | 10 | B | 7th wave | 17 | 6 |
| 25/Aug/2023 | 10 | A | 7th wave | 17 | 6 |
| 26/Aug/2023 | 10 | A | 7th wave | 15 | 6 |
